# What Can Lexical Tone Training Studies in Adults Tell Us about Tone Processing in Children?

**DOI:** 10.3389/fpsyg.2018.00001

**Published:** 2018-01-23

**Authors:** Mark Antoniou, Jessica L. L. Chin

**Affiliations:** The MARCS Institute for Brain, Behaviour and Development, Western Sydney University, Sydney, NSW, Australia

**Keywords:** lexical tone, training, acquisition, individual differences, adults, children

## Abstract

A growing number of studies on the acquisition of lexical tone by adult learners have revealed that factors such as language background, musical experience, cognitive abilities, and neuroanatomy all play a role in determining tone learning success. On the basis of these findings, it has been argued that the effectiveness of tone learning in adulthood depends on individual differences in these factors. However, it is not clear whether similar individual differences play an analogous role in tone learning in childhood. Indeed, relatively few studies have made comparisons between how adults and children learn lexical tones. Here, we review recent developments for tone learning in both adults and children. The review covers tone training in a range of contexts, including in naive listeners, in native speakers of other tone languages, in listeners with varying levels of musical experience, and in individuals with speech and hearing disorders. Finally, we discuss the parallels between adult and child tone learning, and provide recommendations concerning how findings in adult tone training can provide insights into tone learning for children by accommodating the needs of individual learners.

## Perception of Tones

In recent years, researchers have developed a sophisticated understanding of lexical tone acquisition in adults. Long-term experiences such as language and music exert a persistent influence that shapes the perception of lexical tone, and has implications for subsequent training and acquisition of non-native tone contrasts. To date, the vast majority of this work has been conducted on adults. In this review, we summarize the adult tone research literature and highlight several of the emerging themes that may guide future research and subsequently elucidate our understanding of tone processing in children.

Many of the world’s languages use pitch patterns called lexical tones as a contrastive feature. These tone languages, such as Mandarin, Thai, and Cantonese, use lexical tones to differentiate the meanings of words. For example, the Mandarin syllable /ma/ can mean ‘mother,’ ‘hemp,’ ‘horse,’ or ‘scold’ depending on which of the four Mandarin tones are used. Similar pitch variations are not lexically meaningful in non-tone languages such as English. Such language differences have been shown to have a profound effect on the processing of lexical tones. For instance, native Mandarin Chinese listeners show an advantage when identifying tones (Gottfried and Suiter, 1997), and also show evidence of strong categorical perception of Mandarin Chinese tones, whereas English listeners do not (Xu et al., 2006; Wu and Lin, 2008). Taiwan Mandarin listeners perceive tones quasi-categorically but French listeners perceive tones psychophysically rather than as contrastive linguistic categories (Hallé et al., 2004). Native listeners of Mandarin, Cantonese, and German show comparable boundaries for rising and falling tone continua, but the Mandarin and Cantonese speakers were more categorical in their discrimination than the Germans who were more psychophysical (Peng et al., 2010). These native language advantages have also been observed in neuroscientific investigations of tone processing (Gandour et al., 2000). Native tone listeners show an advantage in cortical processing (Wong et al., 2004; Chandrasekaran et al., 2007, 2009b), more faithful and robust subcortical encoding of tone (Krishnan et al., 2005), and also potentially left-hemisphere specialization (Wang et al., 2004). Tone languages also vary considerably in the size and composition of their tone inventories, and this has consequences for the perception of non-native tones.

One possible explanation for how language background shapes tone processing is that tone and non-tone language speakers rely on different acoustic cues when discerning lexical tones. Specifically, language experience has been shown to shape perception of pitch such that listeners attend to pitch information that is meaningful in their native language (Braun and Johnson, 2011), thereby affecting perception of pitch in non-native languages (Schaefer and Darcy, 2014). For native Mandarin speakers, the primary cue for tone contrasts is the fundamental frequency (F0) contour (Xu, 1997; Liu and Samuel, 2004); conversely, native English speakers appear to rely on absolute height when differentiating Mandarin tone contrasts (Wang et al., 2003a). Further, Chinese listeners integrate consonantal and tonal information, whereas English listeners perceive tones and consonants as dimensions that may be separated (Lin and Francis, 2014).

Evidence is mixed concerning whether tone language speakers have an advantage when perceiving tones in a non-native language. On the one hand, numerous studies have shown that prior tone language experience improves subsequent perception of non-native tones. For example, Cantonese listeners outperformed Mandarin and English listeners on Cantonese tones, and Mandarin listeners outperformed Cantonese listeners who in turn outperformed English listeners on Mandarin tones (Lee et al., 1996; Schaefer and Darcy, 2014). Further, native Mandarin speakers identified Mandarin tones more accurately than non-native speakers of varying Mandarin experience (ranging from 1 to 4 years) and this pattern remained the same under talker variability or increased noise (Lee et al., 2010). Moreover, as experience with a tone language increases, so does the ability to correctly perceive contextual variations that affect tone identity and fine-grained acoustic differences between certain tone contrasts. This is important because tone identification critically depends on the preceding context (Moore and Jongman, 1997), and the ability to discriminate acoustically similar tones as well as the complex phonological relationship between them (Hao, 2012). Inexperienced listeners tend to assimilate second language (L2) tones to native language (L1) tones with the most similar acoustic properties (i.e., F0 height and contour) whereas experienced listeners are also sensitive to higher order phonological tone changes (Wu et al., 2014). Native tone language experience also facilitates perception of non-native tones when spoken by multiple talkers (Chang et al., 2017).

However, there is also evidence that prior tone language experience may interfere with non-native tone perception. For instance, Cantonese listeners were constrained by their native phonology (e.g., the phonemic status and the F0 patterns of Cantonese tones) on a task using Mandarin tones, but similar constraints were not observed in Japanese or English listeners, suggesting that the native phonological system may interfere when perceiving non-native tones (So and Best, 2010). Similarly, So (2005) found that native Cantonese speakers encountered more difficulty than native Japanese speakers when distinguishing between Mandarin tones 1 and 4, as well as tones 2 and 3. Tone language experience may also interfere with non-speech tone processing. Mandarin listeners were hindered in their perception of flat and falling pitch contours of non-speech stimuli and misidentified these stimuli more often than did English listeners (Bent et al., 2006). In summary, language experience shapes perception of native, non-native and non-speech tones. Therefore, differing language experiences are likely to have a profound effect on subsequent tone learning in a foreign language. Bearing this in mind, let us next turn to training studies that have attempted to teach learners with varying levels of tone language experience to discern unfamiliar, novel tones.

## Tone Training in Naive Listeners

It is well-established that the perception of non-native lexical tone contrasts is difficult for adult L2 learners (Burnham and Francis, 1997; Wang et al., 1999, 2003a; Wayland and Guion, 2004), particularly for those whose L1 does not make use of pitch height and movement to signal changes in word meaning. Nevertheless, speech training can improve tone identification accuracy in such listeners who possess no prior tone language experience (Wang et al., 1999). Neuroscientific studies have confirmed that tone training paradigms result in reliable changes in the learners’ brains (Wang et al., 2003b). For example, successful versus unsuccessful learners of a tone speech training program showed different patterns of brain activation following training (Wong et al., 2007a). In a similar training study, individuals who were better at learning non-native tones showed larger repetition suppression in the left inferior frontal gyrus (Asaridou et al., 2016). Brain plasticity changes have also been observed in the auditory brainstem following short term tone training (Song et al., 2008). Subsequent studies have examined the effectiveness of different training types, and the factors that are likely to contribute to successful learning outcomes, including the learnability of tones, more generally.

Tone training has also been investigated in studies that have examined naive learners’ abilities to track statistical regularities in the environment. Distributional learning experiments manipulate these statistical regularities to induce learning of one or two categories by presenting unimodal or bimodal stimulus distributions, respectively. In one such study, Australian English listeners were trained on a Thai tone contrast, and those who were exposed to a bimodal distribution learned better than those exposed to a unimodal distribution, but this bimodal advantage only emerged when the task required that they attend to the stimuli (i.e., the bimodal advantage not observed for passive learning), suggesting that auditory attention is necessary for tracking statistical regularities (Ong et al., 2015). In a subsequent study comparing Mandarin native listeners to Australian English musicians, the Mandarin natives showed distributional learning as above, but Australian English musicians benefitted from both bimodal *and* unimodal exposure (Ong et al., 2017).

Tone word learning studies require participants to accurately perceive lexical tones in order to differentiate newly learned words, often containing the same segments. Although it has been suggested that listeners may be more aware of phonological segments than tones (Burnham et al., 2011), a word learning study by Antoniou and Wong (2016) found that English listeners were more successful at learning to map non-native tone contrasts to meaning than a non-native prevoiced-unaspirated voicing contrast, suggesting that tone contrasts may be easier to acquire than some consonantal distinctions (possibly because the learners’ native language interfered with the learning of a non-native voicing contrast). In another study, non-tone language speakers were able to learn a vocabulary of non-native tone words, although considerable individual differences were observed, and tone word learning performance correlated with pretraining pitch perception ability and music experience (Wong and Perrachione, 2007). In a novel word learning experiment, Mandarin–English bilinguals were better than English monolinguals at using tone to identify novel words, and their performance correlated with degree of Mandarin dominance (Quam and Creel, 2012). Other word learning studies have investigated whether different elements of training paradigm design can boost tone learning. For English listeners, it has been suggested that orthography (e.g., tone marks) leads to better learning outcomes (Showalter and Hayes-Harb, 2013). Additionally, instructing native English speakers to focus on pitch direction, rather than pitch height, improves performance on a tone categorization task (Chandrasekaran et al., 2016).

A growing number of studies are taking into account individual differences among learners by examining pretraining abilities (i.e., sensitivity to tones), and memory availability, and examining how these interact with training paradigm designs such as those mentioned above. In a study involving native English speakers, pitch identification ability was a better predictor of performance on a Mandarin word learning task than musicality, language aptitude, or general cognitive ability and predicted generalization to new talkers (Bowles et al., 2016). High stimulus variability improved learning for learners with strong pretraining abilities but hindered the performance of low-aptitude individuals (Perrachione et al., 2011; Sadakata and McQueen, 2014). This suggests that learners with differing pretraining abilities will likely benefit from tailored training approaches that take these individual differences into account rather than one-size-fits-all approaches. Consistent with this idea, in a comparison of learners with high and low pretraining pitch sensitivity, learners with low pretraining pitch sensitivity showed the greatest improvements when lexical pitch pattern training preceded lexical training (Ingvalson et al., 2013).

In a study examining older adults, learning performance was best predicted by declarative memory capacity rather than baseline sensitivity for pitch patterns or working memory capacity (Ingvalson et al., 2017). This finding suggests that older adults may benefit from non-native speech training paradigms that have been tailored to the needs of individual learners, and the variables that predict performance differ across the lifespan (i.e., pitch pattern sensitivity in young adulthood vs. declarative memory capacity in older adulthood). Given that training older adults demonstrably relies on different predictors of tone learning performance than young adults (likely due to the effects of age-related cognitive decline in older adulthood), training children will also likely require a different set of predictors because it is during the course of childhood that the foundations of cognitive abilities are established. The crucial point is that training studies involving children should also measure pretraining abilities with a view to tailoring training to maximize learning outcomes.

This summary of the field on tone training in naive learners suggests that those with better pretraining abilities will benefit most from training. Who is a good versus poor learner appears to be dependent on perceptual and neurophysiological differences. It is also likely that differences can be accounted for by differences in cognitive ability (MacDonald, 2008; Majerus et al., 2008) or variations in language background (Iverson and Evans, 2009) although these studies have not examined tone training. Given the importance of learners’ pretraining abilities, training paradigms must take into account individual differences among learners in order to produce the best training outcomes. Further, it is likely that the contribution of such factors is likely to vary across the lifespan. A fruitful avenue for future research would be to examine the contribution of such individual differences in children, and how these contributions vary as the child develops.

## Non-Tonal L1 Speakers Learning a Tonal L2

We have thus far reviewed tone training in naive listeners with no tone language experience. Other studies have examined how varying degrees of L2 experience with a tone language affects processing and learning of lexical tones. First, let us look at studies of non-tonal L1 speakers who are actively acquiring a tone language as their L2.

Non-tonal L1 speakers who learn a tonal L2 provide a fascinating opportunity to examine the flexibility of the human perceptual system to attend to acoustic cues that serve a critical linguistic function in only one of their languages. A tone language learner may rely on either F0 height or direction when perceiving pitch, depending on their language background. L1 English–L2 Mandarin speakers, who rely on F0 height in their L1 and F0 direction in their L2, were exposed to four of the six Cantonese tones, with English monolinguals and L1 Mandarin speakers serving as the control groups. L2 learners, as well as the Mandarin controls, were significantly better at discriminating the contour–level tone pairs than the level–level tone pairs. This suggests that L2 experience increased L2 learners’ sensitivity to F0 direction in the perception of unfamiliar tones (Qin and Jongman, 2016). It should be noted that the Mandarin controls performed significantly worse than the other groups when perceiving level–level tones, suggesting that L1 tone experience did not provide a perceptual advantage for all tone pairs. Similarly, Mandarin speakers outperformed L1 English–L2 Mandarin speakers who in turn outperformed English monolinguals in non-linguistic and Mandarin discrimination tasks and a pitch-shift task. Further, discrimination of musical and Mandarin tones were correlated (Ning et al., 2014). Japanese-speaking Mandarin learners of elementary and intermediate proficiency levels were exposed to utterances in quiet and noisy listening conditions. When recognizing L2 Mandarin speech, the Japanese-speaking Mandarin learners were affected by their L2 proficiency, the semantic context, F0 contours, and noise (Zhang et al., 2016). In another study, students in an introductory Chinese language course were trained to identify tones via three different training types. Those who received training with visual pitch contours and pinyin performed better than students trained with visual contours only or with tone numbers and pinyin (Liu et al., 2011). Wang (2013) found that introductory (first semester) learners of Mandarin identified tone 3 most accurately regardless of L1 background (Hmong, Japanese, American English). Hmong speakers perceived Mandarin tones less accurately than the Japanese and English groups, experiencing the most difficulty in perceiving tone 1, but significantly improved in accuracy after training. Japanese speakers did not benefit from pitch accent in their L1, as they performed similarly to the English speakers in accuracy. By the end of training, all groups improved in accuracy, and Hmong and Japanese speakers were neither advantaged nor disadvantaged by their L1 prosodic backgrounds.

Several studies have observed that L1 intonation patterns exert an effect on the perception of non-native tones. For instance, when presented with Mandarin tone continua, Taiwanese listeners were more sensitive to between-category differences than within-category, whereas French listeners were equally sensitive to differences across continuum steps (Hallé et al., 2004). In another study, Mandarin and English native listeners showed different error patterns when perceiving Cantonese tones; the English listeners identified the high rising tone accurately, perhaps because it was perceived as similar to English question intonation, but poorly identified low rising and low falling tones because they did not map onto any native intonation pattern (Francis et al., 2008). The implication of these studies is that non-tone language speakers can map non-native tones onto the intonational contours used in their native language, and this may in turn influence non-native tone processing.

Neural investigations have shown that reliable brain changes follow a semester of Mandarin learning in college (Wang et al., 2003b). In this functional neuroimaging study, American English speakers studying beginner-level Mandarin completed eight sessions of tone training. Locations of activation in the cortex remained the same in pre- and post-training scans, including the left medial frontal gyrus, and bilaterally in the inferior frontal, middle temporal, and superior temporal gyri. Enrichment plasticity was observed in the early stages of L2 learning, shown by the expansion of cortical regions and recruitment of additional cortical areas specialized toward similar language functions, namely within the left superior temporal gyrus and the right inferior frontal gyrus. In sum, these studies demonstrate that even relatively short-term tone language learning (e.g., over a semester) leads to reliable learning advantages in acquiring novel tones, and also results in reliable learning-related brain changes.

## Tonal L1 Speakers Learning a Tonal L2

Other training studies have investigated whether tone language speakers possess an advantage when it comes to learning the tones of an unfamiliar tone language. There is indeed evidence that sensitivity to tones in one language may boost perception of tones in another language (Wayland and Guion, 2004), such that knowledge of a tone language (e.g., Mandarin Chinese) may improve learning of tones in another (e.g., Thai) relative to controls that lack tone language experience (Wayland and Li, 2008). There is also evidence that speakers of tone languages exhibit superior performance in pitch-recognition tasks (Caldwell-Harris et al., 2015). The explanation for such advantages may lie in the native tone language speaker’s ability to attend to the critical acoustic cues that differentiate lexical tones, even in non-native tone languages. For instance, native speakers of Mandarin Chinese show greater perceptual sensitivity to pitch contour differences later in the signal, while English speakers are more sensitive to earlier pitch differences (Kaan et al., 2007). This is consistent with neurophysiological studies that have shown that brainstem mechanisms for pitch encoding, as reflected in pitch-tracking accuracy and pitch strength, are more sensitive in tone (Chinese, Thai) than non-tone (English) language speakers (Krishnan et al., 2010). These differences in brainstem encoding give tone language speakers an advantage in perceiving minute changes in pitch, and may ultimately bear on tone learning outcomes.

Furthermore, native tone language speakers are capable of learning new tone contours in adulthood. Studies examining tone learning in adults have confirmed that language background affects both attentive and non-attentive processing of tone contrasts, but processing of pitch is malleable even in adulthood (Kaan et al., 2008; Chandrasekaran et al., 2009a). Additionally, forming new speech categories that depend on unfamiliar perceptual dimensions (such as lexical tone for non-tone language speakers) results in stronger gamma activity and more coherent alpha-band activity than forming new categories using familiar dimensions (Kaan et al., 2013).

The above evidence suggests that tone language experience brings subsequent tone learning advantages and that the adult brain is capable of learning novel tone contrasts from a foreign tone language. Whether similar advantages arising from prior tone language experience occur in children remains to be seen.

## Tone Learning in Individuals with Musical Experience

Both music and lexical tone place great importance on pitch, and thus a growing number of studies have investigated whether musical expertise results in tone language processing (and ultimately learning) advantages. Experience-dependent bidirectional transfer effects have been observed between speech and music (Chang et al., 2016). On the one hand, musicians show advantages in cortical (Schön et al., 2004; Marie et al., 2011, 2012) and subcortical (Wong et al., 2007b) processing of pitch. On the other hand, tone language speakers show enhanced musical pitch processing (Chandrasekaran et al., 2009a; Bidelman et al., 2013) and more robust brainstem encoding of musical pitch (Bidelman et al., 2011). Musical training has been shown to facilitate lexical tone identification, but the degree of facilitation is modulated by the tone in question and the type of acoustic input (Lee and Hung, 2008).

In terms of training, musical or tone language experience are associated with significantly better non-native word learning proficiency of tone-based words, as compared to individuals with neither musical training nor tone language experience (Cooper and Wang, 2012). Further, the combination of tone language and musical experience did not result in an additive advantage for Thai musicians above and beyond either experience alone. In a separate study, musicians who completed a 2-day training protocol identified pitch contours more accurately than non-musicians, although their pitch contour abstraction ability (to other stimuli) was similar to that of non-musicians (Wayland et al., 2010).

Therefore, musical experience improves pitch encoding and leads to some lexical tone training advantages, although this is modulated by other factors. Similar effects would presumably emerge in children with music experience, although this warrants systematic investigation.

## Tone Training in Individuals with Speech and Hearing Disorders

Tone training has also been investigated in individuals with speech and hearing disorders (e.g., amusia) and hearing impairments (e.g., cochlear implant recipients). A small but growing body of research has examined the congenital disorder amusia (or tone-deafness) that impairs the ability to perceive pitch in language and music (Peretz, 2001; Ayotte et al., 2002). The general finding from this research literature has been that amusic listeners of a non-tone language consistently perform worse than speakers of non-tonal languages when exposed to lexical and non-speech tones. French amusics were poorer than controls at discriminating Mandarin lexical tones, although there was considerable overlap in performance (Nguyen et al., 2009). In another study, French amusics experienced greater difficulty discriminating lexical tones taken from Mandarin or Thai words, and acoustic analyses revealed that amusics relied on cues such as sound duration and intensity to compensate for their pitch perception deficit (Tillmann et al., 2011). British English amusics showed impaired discrimination, identification, and imitation of statements and questions that differed in pitch in the final word (Liu et al., 2010). Further, those amusics with smaller pitch thresholds tended to perceive intonation more accurately. English-speaking amusics were poorer at processing speech sounds (phonological and phonemic awareness), indicating deficits in sound processing that are not restricted to the domain of music (Jones et al., 2009).

Amusics who are native speakers of a tone language are also impaired in their ability to discriminate and identify lexical and non-speech tones. The majority of these studies focus on amusic Mandarin speakers. In one study, Mandarin amusics experienced difficulty identifying and discriminating Mandarin tones, with some participants exhibiting signs of lexical tone agnosia, that is, an inability to distinguish lexical tones (Nan et al., 2010). Interestingly, no analogous deficits were observed in Mandarin tone production, implying that congenital amusia primarily impairs the perception of pitch. Mandarin amusics have also shown poorer performance than controls for tasks relying on pitch sensitivity, but are not impaired when completing tasks involving multiple acoustic cues (Liu et al., 2012a). They have greater difficulty recognizing the pitch direction of discrete tones, rather than gliding tones, a pattern observed for both speech and non-speech stimuli (Liu et al., 2012b). Furthermore, Mandarin amusics were impaired in their perception of both lexical and non-speech intonation patterns (Jiang et al., 2010). Mandarin amusics who had undergone pitch sensitivity training had improved tone identification thresholds for both speech and music, matching the performance of non-amusic controls (Liu et al., 2017).

Cochlear implant recipients are also impaired in their ability to perceive pitch, and this has serious implications for speakers of tone languages. For example, Mandarin-native cochlear implant users scored between 30 and 50% on Mandarin tone recognition tests (Wei et al., 2004). To address this, training regimens have been developed that aim to improve cochlear implant users’ perception of lexical and non-speech tones. Training methods range from training with musical instruments (Yucel et al., 2009) to various computer-assisted training software programs such as Computer-Assisted Speech Training (Fu and Galvin, 2007) and the Melodic Contour Training Program (Lo et al., 2015). Music training has been shown to improve accuracy in musical perception, and also has implications for improving speech (e.g., pitch) processing (Gfeller et al., 2015). Computer-assisted training software offers auditory training for adult cochlear implant recipients, and has been shown to be effective in improving cochlear implant recipients’ speech and music perception (Fu and Galvin, 2007). Melodic contour training has also been effective in improving cochlear implant users’ perception of question/statement intonation and consonants in quiet environments (Lo et al., 2015). In a study of postlingually-deafened adult cochlear implant recipients who underwent 20 h of auditory computer-assisted training over 4 weeks, 6 of the 7 subjects improved in speech recognition performance using electronic-only stimulation and electronic and acoustic stimulation. However, improvements were not observed in those who underwent acoustic-only stimulation (Zhang et al., 2012). These studies suggest that even individuals who have difficulty perceiving pitch (i.e., both amusics and cochlear implant users) may improve their perceptual accuracy following science-based training interventions. Although more research is needed, this body of work provides hope to many affected individuals faced with the challenge of acquiring a tone language under challenging conditions, and paves the way for future interventions involving children with similar conditions.

## Tone Acquisition in Children

The sections of the review thus far have covered studies that have examined tone learning in adults. The remainder of the article will be devoted to covering the work that has examined these abilities in children, and proposing how the field may be advanced by addressing the research questions that have been raised in the adult tone learning literature.

Using discrimination tasks adapted for infants, researchers have begun to understand the timeline of the developmental processes that underpin language development, including the development of sensitivity to lexical tones (Nazzi et al., 1998; Cheng et al., 2013). During the first year of life, infants attune to the elements of their native language and discrimination of non-native language elements deteriorates, in a process termed perceptual reorganization. It is now clear that language-specific speech perception follows a more complex developmental schedule than had been previously thought. Infants first attune to native lexical stress and tone patterns by 5 months of age, then vowels at 6–8 months, consonants at 9–12 months, and phoneme duration at 18 months (Yeung et al., 2013). Studies on tone acquisition in infants have uncovered the developmental window during which perceptual reorganization for lexical tones occurs. In a series of studies, Mattock and colleagues demonstrated that Chinese infants are able to discriminate both Thai lexical tones and non-speech (violin) tones equally well at both 6 and 9 months of age. In contrast, English infants lose their ability to discriminate Thai rising versus falling and rising versus low level lexical tones between 6 and 9 months, but their ability to discriminate non-speech tones is unaffected, suggesting that lexical tone perception in the first year of life is critically dependent on the native language environment (Mattock and Burnham, 2006; Mattock et al., 2008). Additionally, infants develop sensitivity to their native tone distinctions in an asymmetric fashion (Harrison, 2000).

Given that infants’ sensitivity to non-native lexical tones diminishes over the first year of life, this raises questions regarding the specific nature of the resulting perceptual constraint and how it interacts with language experience. In a distributional learning study, 5-, 11-, and 14-month-old Dutch infants were familiarized with a unimodal or bimodal distribution of high-level versus high-falling Mandarin tones; the 5-month-olds discriminated both, the 11-month-olds discriminated the bimodal distribution, but the 14-month-olds were not able to discriminate either (Liu and Kager, 2017b). Subsequent studies have demonstrated that although infants’ ability to discriminate Mandarin high-level versus high-falling tone contrasts diminishes by 9 months of age, their sensitivity rebounds by 18 months (Liu and Kager, 2014), and perhaps even sooner in the case of bilinguals (Liu and Kager, 2017a).

Indeed, studies involving bilingual infants supplement findings from monolinguals and demonstrate that early experience with multiple languages may improve perceptual flexibility. In one such study, 7.5-month-old Mandarin–English bilingual infants were able to recognize English words that were matched in pitch and Mandarin words matched in tone. 9-month-olds recognized English words that were mismatched in pitch or Mandarin words that contrasted in tone. By 11 months, however, infants had learned to correctly recognize English words that were pitch-matched and -mismatched, but only recognized tonal matches in Mandarin (Singh and Foong, 2012). Interestingly, a perceptual shift has been observed in Mandarin–English bilingual children such that 2.5–3.5-year-old toddlers’ word recognition abilities are more affected by deviations in lexical tones than in segments, but 4–5-year-old preschoolers are more affected by deviations in segments than in tones (Singh et al., 2015). This observation is consistent with evidence that when 2.5–3.5-year-old Mandarin toddlers and 4–5-year-old preschoolers were presented with Mandarin words where intonation (question/statement) or tone (rising/falling) were manipulated, toddlers made errors due to intonation, whereas preschoolers recognized tone words regardless of intonation (Singh and Chee, 2016).

Further changes continue to emerge as a result of long-term experiences as the child enters school age and progresses toward adolescence and adulthood. In Cantonese children, tone recognition improves from ages 4 to 6, and from ages 6 to 10, at which point children perform as accurately as adults (Ciocca and Lui, 2003). Mandarin children tend to produce tones less accurately than adults, based on the ratings of native judges (Wong et al., 2005). Further, children perceived the level, rising, and falling tones accurately, but struggled to perceive and produce the dipping tone (tone 3). Children identified Mandarin tone 4 least accurately and tone 1 consistently, and they mostly confused Mandarin tones 2 and 3, followed by tones 1 and 4 (Li et al., 2017). The composition of the native tone inventory also shapes children’s perception of non-native tones. For instance, Cantonese has a six-tone system, whereas Mandarin only has four tones, and it was observed that Cantonese-speaking first graders performed better in tone awareness tasks than their Mandarin-speaking counterparts (Chen et al., 2004). Moreover, long-term experience with music predicts better tone identification in Italian-speaking children between the ages of 6 and 8, but it does not predict phonological identification (Delogu et al., 2010).

In sum, the available evidence suggests that similar long-term experiential effects may be observed in children as in adults. Native language background, complexity of the native tone inventory, and prior music experience all contribute to tone processing in children. It is not yet clear, however, how such experiences interact with the emergence of tone processing abilities in children, and which factors take precedence at which timescales of development.

## Tone Training in Children

Tone training studies in young children have revealed that they are initially sensitive to acoustic differences between lexical tones, but those children from non-tone language backgrounds gradually learn to ignore such pitch differences as lexically relevant. Infant studies adapt word learning and familiarization tasks so that they are age-appropriate in their attentional and task demands, including how responses are measured and by employing a reduced number of trials. Singh et al. (2008) familiarized 7.5- and 9-month-old English infants with words and observed that while the 7.5-month-olds recognized words with matching pitch contours, the 9-month-olds treated words with mismatched pitch contours as equivalent. In another study, 14-month-old English infants learned labels for two novel objects that were differentiated by differing pitch contours, whereas 17- and 19-month-olds were unable to learn the picture-label pairings despite being able to differentiate the pitch contours in a separate task (Hay et al., 2015). This suggests that 14-month-olds are flexible learners when it comes to perceiving sounds that distinguish words, but for 17–19-month-olds with a non-tonal native language lexical tone is no longer treated as relevant for differentiating words. In another study, English-speaking adults and 2-year-olds learned a new word that would later undergo either a phonemic or pitch change. Changes in vowel-quality impaired word recognition, but changes in pitch contour did not, indicating that by the age of two, English-learning children disregard variations in pitch when recognizing words (Quam and Swingley, 2010). These studies suggest that experience with a non-tonal language constrains perceptual flexibility and integration of lexical tone into the learning of novel words. Interestingly, while this seems to be the case for monolingual infants, there is evidence that bilingual infants remain sensitive to non-native lexical tone differences longer than monolingual infants of the same age and are able to use non-native pitch contours to differentiate newly learned words even at 17–19 months but not at 22 months (Graf Estes and Hay, 2015).

Studies on bilingual infants suggest that bilingualism leads to certain perceptual advantages that may aid tone learning. At 12–13 months of age, Mandarin–English bilingual infants were able to use tone to differentiate newly learned words in Mandarin (but not English), whereas Mandarin monolingual infants were unable to learn the words until 17–18 months even though they were capable of discriminating the tones (Singh et al., 2016). At 18 months, bilingual children are predisposed to process tone as lexically relevant regardless of their native language, but at 24 months, only tone-language-speaking children continue to do so (Singh et al., 2014). These findings suggest that bilinguals remain perceptually flexible for longer than monolinguals (see Graf Estes and Hay, 2015; Hay et al., 2015) and may be sensitive to a wider variety of acoustic dimensions when learning label-object mappings to differentiate novel words at this age. Language-specific sensitivity continues to develop beyond toddlerhood. Mandarin–English bilingual 3 to 4- and 4 to 5-year-olds completed a word learning experiment and were presented with words that were matched or mismatched in tone and presented in English or Mandarin contexts. The 4–5-year-old preschoolers were able to process tone as lexically meaningful in Mandarin and disregard it in English, but the 3–4-year-olds could not (Singh and Quam, 2016).

Studies on children from tone language backgrounds have shown that while they are capable of learning novel tone categories, their perceptual performance continues to develop and improve throughout childhood as they grow. For instance, both 2- and 3-year-old monolingual Mandarin Chinese children struggled to recognize words in the presence of vowel and tone variation, but sensitivity to these features were age-dependent. Specifically, only 2-year-olds performed poorly in word recognition in response to tone variation, while 3-year-olds showed insensitivity to tone variation and were able to use tones to learn new words, although tone 3 words were most difficult to learn (Ma et al., 2017). Further, tone learning abilities continue to develop throughout childhood. 6-, 10-, 14-, and 19-year-olds completed computerized Mandarin training in six 40-min training sessions over a period of 2 weeks, and children at all ages showed significant improvement, but not controls (who played computer games for the same time) (Wang and Kuhl, 2003). In a study investigating the perceptual abilities that correlate best with language development (as indexed by a narrative story-retelling task), Cantonese school-aged children in grades 1–6 completed a series of AX discrimination tasks that assessed their temporal and pitch-based auditory abilities. Temporal abilities were measured using a music rhythm task in which pairs of melodies were presented and some trials contained a change in rhythm caused by having a musical note occur in a different location. Pitch abilities were measured using a pitch pattern perception task involving non-speech pitch contours, and a music scale task where some melodies contained a musical note that differed by four semi-tones. Both temporal and pitch abilities correlated with language development, but only pitch abilities (i.e., performance on pitch pattern perception and music scale tasks) explained unique variance in narrative ability scores after age (Antoniou et al., 2015). This suggests that pitch abilities play a crucial role in linguistic development of tone-language-speaking children. Further, children in grades 5 and 6 did not match the level of performance of adults in terms of their temporal and pitch abilities, suggesting that sensitivity to these dimensions continues to develop into adolescence.

Other than studies that have looked at training using speech stimuli, there is also evidence that music training in childhood can boost pitch processing. In one such study, 8-year-old Portuguese children who completed music training for 6 months improved their reading ability and pitch discrimination in speech, but those who completed painting training for the same amount of time did not (Moreno et al., 2009). These findings are supported by the observation that 8-year-old children with several years of music experience showed an advantage in detecting subtle pitch deviations both in musical notes and lexical tones (Magne et al., 2006). These results reveal positive transfer effects from music training to speech processing in children, analogous to the long-term experiential effects of musicianship on speech processing observed in adult musicians.

These studies provide useful starting points, but many questions remain concerning the tone learning abilities of children. Very little attention has been paid to individual differences in child learners and thus it is not clear what makes some children more successful learners than others. Useful variables to consider include native language experience, pretraining tone sensitivity, prior music experience, working memory availability, and neurophysiological differences. By isolating the combination of factors that matter most for successful tone learning, it may ultimately be possible to tailor tone training proactively to the needs of individual child learners, including those with speech and communication disorders or hearing impairments, which we will now review.

## Tone Processing in Children with Hearing Impairments, Speech and Developmental Disorders

A small number of studies have examined children with speech and developmental disorders or hearing impairments. These studies offer some clues concerning how the effects of such conditions may be ameliorated by behavioral interventions. A number of these studies have examined tone language-learning children with cochlear implants. Children with cochlear implants performed significantly worse than normal hearing controls in Cantonese tone perception, and their tone perception developed in a pattern differing from normal hearing children (Lee et al., 2002). Additionally, children with profound hearing impairment tend to produce tones less accurately than those with mild hearing loss (Cheung et al., 2014). These observations are consistent with findings from the adult literature.

There is also some promising evidence that children with cochlear implants benefit from training interventions. Children with newly-fitted cochlear implants participated in a family-oriented music training program that consisted of pitch, note, and rhythm discrimination exercises on an electric keyboard. Children who underwent musical training had more interest in music and, after 24 months, showed greater development in all areas of music perception. However, only modest improvements were observed for speech perception in the musically-trained children relative to controls (Yucel et al., 2009). In another study, Mandarin-speaking children with cochlear implants completed melodic contour identification training for 10 weeks. Performance improved after 4 weeks of training, and no performance decline was observed at the 8-week follow-up (Fu et al., 2015). Computerized speech training delivered for half an hour per day, 5 days per week, for 10 weeks improved vowel, consonant, and tone recognition performance of hearing-impaired children and these benefits were maintained 2 months after the cessation of training (Wu et al., 2007). These studies suggest that although cochlear implants are limited in their capacity to effectively provide F0 information, training interventions can teach children who use cochlear implants to attend to other available acoustic cues (e.g., temporal envelope cues) and improve perception of lexical tones.

Recent studies have begun to shed light on how tone processing is influenced by other speech and developmental disorders that affect children. Work by Wong et al. (2009) has demonstrated that poor tone identification in children with specific language impairment is not only affected by vocabulary knowledge, but some children also had deficits in pitch processing and/or pitch categorization. Moreover, a study comparing Chinese children with dyslexia to chronological-age controls and reading-level controls found that children with dyslexia had a later developmental ceiling, and their lexical tone awareness distinguished them from typically developing children across the primary school years. A perceptual training intervention was employed with the goal of improving lexical tone awareness and character naming in dyslexic children. Only second-grade children improved in both aspects, in comparison fourth-grade children showed improved performance in lexical tone awareness only (Wang et al., 2017). This suggests that children with dyslexia may benefit from perceptual training but more research is needed to maximize any training-related outcomes. Furthermore, a study involving Mandarin children with autism demonstrated that they were able to comprehend and discriminate tones equivalently to their typically developing peers, but they made different errors when presented with the tone 2–3 contrast. Additionally, children with both autism and significant language problems treated nonce word stimuli like pure tone stimuli, thus showing unstable abstract representations of tones (Lu, 2016). These studies demonstrate that perception of lexical tones in one’s native language may be affected by a variety of speech and developmental disorders. Encouragingly, there are early indications that perceptual training interventions may be effective in improving lexical tone perception in some of these populations, although further research is needed.

Although great strides have been made in developing a detailed understanding of how children attune to the native tone system, work on the disorders that affect lexical tone processing is still in its infancy. Encouragingly, there are already signs that children with speech and developmental disorders or hearing impairments benefit from training interventions designed to improve tone processing. Future work should strive to improve the efficacy of such interventions.

## Conclusion

In this review, we have summarized recent developments in the tone learning literature for both adults and children. The literature covered addresses tone training across a range of adult learners differing in their prior language experience from naive listeners, to L2 tone language learners, to native speakers of other tone languages, to those with varying levels of musical experience, as well as individuals with speech and hearing disorders. Below we relate each of these factors to the most relevant research conducted in infants and children.

Evidence from speech perception, word learning, and neurophysiological studies on adults has demonstrated that prior language experience exerts a profound and persistent influence on the processing of non-native lexical tones. Native tone language experience may aid processing of similar tone contrasts, but may also in some cases interfere with the processing of non-native tones (e.g., when two non-native falling tones are perceptually assimilated to a single native falling tone category). Additionally, speakers of non-tone languages may benefit from similarities to native intonational patterns (Hallé et al., 2004). In general, fewer studies have been conducted on infants and children than adults, and the evidence is largely in congruence with the data on adults. Native language tone processing advantages have been observed in infants (Mattock and Burnham, 2006), toddlers (Singh and Chee, 2016), and children (Ciocca and Lui, 2003). Benefits have also been observed for non-native tone training (Wang and Kuhl, 2003), although even school-aged children do not match adults in their pitch perception abilities (Antoniou et al., 2015), suggesting that sensitivity to tones continues to develop throughout childhood and into adolescence. Studies on bilingual infants have advanced our understanding of the consequences of bilingualism by revealing that they are flexible perceivers, able to integrate non-native tones earlier and for longer than age-matched monolingual peers. Future work is needed to explore the conditions that give rise to such bilingual advantages in perceptual flexibility including language pairings (L1 tone–L2 non-tone language vs. L1 tone–L2 tone language vs. L1 non-tone–L2 non-tone language), intonational patterns present in the child’s known languages, and mapping between known languages and the target language. The paucity of neurophysiological studies on infants’ processing of lexical tone also provides fertile ground for significant contributions in knowledge to be made.

Aside from native language experience, musicianship is another long-term experience that profoundly alters processing of pitch both in speech and non-speech stimuli. While research on the experiential effects of music on adults is growing rapidly, research into the effects of musical training in childhood on lexical tone processing is still in its infancy; however, the few studies that have been conducted have reported that musical training is beneficial for pitch processing (Magne et al., 2006; Moreno et al., 2009).

Several studies on adults present converging evidence that pretraining perceptual abilities account for word learning performance (Perrachione et al., 2011; Sadakata and McQueen, 2014). Further, pretraining perceptual abilities can be used to tailor training to maximize learning outcomes (Ingvalson et al., 2013). Research on infants and children has neglected such pretraining individual differences. Several studies have reported large degrees of variation in young children, but the underlying factors that account for this variation are not yet understood. Thus, there is still much to learn about individual differences in lexical tone processing, especially in individuals experiencing communicative difficulties.

Studies on speech and hearing disorders in adults have revealed that several conditions may lead to difficulties in lexical tone processing. Adults with congenital amusia are impaired in their ability to identify and discriminate lexical tones (Peretz, 2001; Ayotte et al., 2002). Similarly, adults with cochlear implants have difficulties correctly identifying lexical tones (Wei et al., 2004). Encouragingly, both conditions have been shown to respond to training interventions designed to improve pitch processing. There have not been many studies examining effects of speech and hearing disorders on lexical tone processing in children. Nevertheless, there are some positive indications that children with cochlear implants benefit from training to improve the accuracy with which they perceive pitch (Fu et al., 2015) and appreciate music (Yucel et al., 2009) possibly by teaching children with cochlear implants to attend to other available acoustic cues.

In sum, research into the development of sensitivity to lexical tone has revealed that perception, word learning, and subsequent language development follow a complex schedule that is influenced by long-term experience such as active language exposure and bilingualism. In comparison, work on adults has revealed long-term experiences such as language background and musicianship greatly affect processing of non-native lexical tones, that short-term training can improve non-native tone processing, and that individual differences among learners may predict tone learning outcomes. Exploring the interaction of these factors in childhood tone acquisition and tone learning will advance the field of infant and child lexical tone processing and lead to improved learning and interventions for those encountering difficulties in processing lexical tones.

## Author Contributions

All authors listed have made a substantial, direct and intellectual contribution to the work, and approved it for publication.

## Conflict of Interest Statement

The authors declare that the research was conducted in the absence of any commercial or financial relationships that could be construed as a potential conflict of interest.
